# Pathological Features and Molecular Phenotype of MMTV Like-Positive Feline Mammary Carcinomas

**DOI:** 10.3390/ani11102821

**Published:** 2021-09-27

**Authors:** Francesca Parisi, Luisa Vera Muscatello, Prospero Civita, Francesca Lessi, Michele Menicagli, Francesca Millanta, Barbara Brunetti, Cinzia Benazzi, Giuseppe Sarli, Giulia Freer, Mauro Pistello, Chiara Maria Mazzanti, Alessandro Poli

**Affiliations:** 1 Department of Veterinary Sciences, University of Pisa, Viale delle Piagge n. 2, 56124 Pisa, Italy; francesca.parisi@vet.unipi.it (F.P.); francesca.millanta@unipi.it (F.M.); 2 Department of Veterinary Sciences, University of Bologna, Via Tolara di sopra n. 43, 40064 Ozzano dell’Emilia, Italy; luisaver.muscatello2@unibo.it (L.V.M.); b.brunetti@unibo.it (B.B.); cinzia.benazzi@unibo.it (C.B.); giuseppe.sarli@unibo.it (G.S.); 3 School of Pharmacy and Pharmaceutical Sciences, College of Biomedical and Life Sciences, Cardiff University, Cardiff CF14 4EP, UK; CivitaP@cardiff.ac.uk; 4 Fondazione Pisana per la Scienza Onlus, Via Ferruccio Giovannini n. 13, 56017 San Giuliano Terme, Italy; f.lessi@fpscience.it (F.L.); m.menicagli@fpscience.it (M.M.); c.mazzanti@fpscience.it (C.M.M.); 5 Department of Translational Research and New Technologies in Medicine and Surgery, University of Pisa, Via Savi n. 10, 56126 Pisa, Italy; giulia.freer@unipi.it (G.F.); mauro.pistello@unipi.it (M.P.)

**Keywords:** cat, mammary tumour, molecular profile, mouse mammary tumour virus-like (MMTV-like)

## Abstract

**Simple Summary:**

Mouse mammary tumour virus-like (MMTV-like) is suspected to be involved in human breast cancer and feline mammary carcinomas (FMCs). We previously reported the identification of MMTV-like sequences and viral protein in six of 78 FMCs collected in Tuscany, Italy. To corroborate this finding, FMCs samples collected from a different geographic area were investigated. MMTV-like sequences and p14 protein were identified in three of 24 FMCs collected at the University of Bologna, one tubular carcinoma, one tubulopapillary carcinoma and one ductal carcinoma. All the examined FMCs from Pisa and Bologna were submitted to immunohistochemistry for molecular phenotype characterization. Of the nine positive FMCs, six were basal-like and three luminal-like. This study highlights the presence of MMTV-like sequences and protein in FMCs of different geographic areas. The characterization of molecular phenotype could contribute to understand the possible role of MMTV-like virus in FMC biological behaviour.

**Abstract:**

In the last few years MMTV-like nucleotide sequences were detected in some feline and canine mammary tumours. Due to the confirmed role of cats in the epidemiology of the MMTV-like virus, the aim of this study was to investigate the main pathological features of positive feline mammary carcinomas (FMCs). Twenty-four FMCs were collected at the University of Bologna, submitted to laser microdissection and analysed by nested fluorescence-PCR using primer sets specific for MMTV *env* sequence. For immunohistochemistry, an antibody against MMTV protein 14 (p14) was used. MMTV-like sequences were detected in three out of 24 FMCs (12.5%), one tubular carcinoma, one tubulopapillary carcinoma and one ductal carcinoma. All PCR-positive tumours were also positive for p14. Multiple nucleotide alignment has shown similarity to MMTV ranging from 98% to 100%. All the 102 examined FMCs were submitted to immunohistochemistry for molecular phenotyping. Of the nine MMTV-like positive FMCs, six were basal-like and three luminal-like. Our results demonstrate MMTV-like sequences and protein in FMCs of different geographic areas. Molecular phenotyping could contribute to understand the possible role of MMTV-like virus in FMC tumor biology.

## 1. Introduction

Although the risk factors for mammary tumors in feline species are well established, recent evidence indicates that viruses may have a role in the process of tumorigenesis. The mechanisms of viral tumorigenesis are still unclear, but different hypothesis have been proposed and several studies are focusing on understanding them. Feline leukemia virus (FeLV) and feline immunodeficiency virus (FIV) are the most common viruses in feline species and infect domestic cats worldwide. Even if the two viruses are associated with numerous generally chronic disorders, neoplastic transformation of tissues of FIV/FeLV positive cats may occur due to immunomodulation [1]. A cat with a FeLV positive feline mammary carcinoma associated with uterine cystic endometrial hyperplasia is described by Yoo and Kim [2] suggesting that this case was related to immune decrease by FeLV infection.

Since the 1990s a large amount of scientific evidence suggests a possible existence of a human virus similar to mouse mammary tumour virus (MMTV). MMTV is a betaretrovirus known to cause mammary carcinomas and lymphomas in susceptible mice. It is transmitted exogenously from infected mothers to the suckling pups: through the milk, the viral particles reach the intestinal epithelium of pups where they infect dendritic cells and B lymphocytes in Peyer’s patches of the gut [3,4,5]. The Sag antigen is then presented to the CD4+ T cells that proliferate and produce cytokines, stimulating and recruiting additional dendritic cells, B cells, and T cells. The mammary gland is a site of passage of normal lymphocytes; here, MMTV-infected lymphocytes carry the virus to mammary glandular epithelial cells, target cells for the virus [5]. The long period of latency between the ingestion of infected milk and mammary tumor development suggests that MMTV is a non-acute transforming retrovirus that causes the dysregulation of host gene expression through the integration of its provirus into the host genome [4,5,6]. Proviral DNA must be inserted into the cell DNA and cell division is required to fix the mutation. The insertion results in proto-oncogene deregulation and a higher expression of encoding genes [7]. Members of four cellular gene families have been shown to be rearranged by MMTV integrations: Wnt, Fgf, Notch gene families and the gene encoding the p48 component of eucaryotic translation initiation factor-3 (elF-3p48) [8]. It seems likely that some of the mutations induced by MMTV, and the signaling pathways in which these target genes take part, will be relevant to the progression from preneoplastic to malignant lesion [9]. This concept has gathered greater support due to the evidence that the human orthologs of MMTV common integration sites have been found to be deregulated and/or mutated in human breast cancers. However, indirect mechanisms of MMTV associated tumorigenesis are also hypothetically possible. For instance, and according to other sources, MMTV could be responsible for the activation of an immunoreceptor tyrosine-based mechanism that could suppress apoptosis. Furthermore, other studies proposed different possible indirect mechanisms for the MMTV associated tumorigenesis, suggesting the idea that the infection with a MMTV-like virus could lead to the activation of latent human DNA viruses involved in human breast cancer, such as Epstein–Barr virus (EBV) or human papillomavirus (HPV), as HIV does with Kaposi’s sarcoma, or it could facilitate infection with a second virus involved in human breast cancer [10].

The hypothesis that a virus can be etiologically involved in sporadically occurring breast cancer in humans is based on the presence of MMTV-like sequences in these tumours sharing over 95% identity with MMTV [11,12,13,14]. More recently, an elegant study published by Lessi and co-workers [15] has also shown that MMTV-like betaretrovirus linked to breast cancer has existed in humans at least since the copper age. Besides, in mice and humans, the association between mammary tumours and a MMTV-like virus it has been also demonstrated for other animal species. The first evidence that MMTV could infect feline cells came in 1976. Until then, the virus had only been found in human milk as well as in mice [16]. In the same year, Vaidya et al. [17] confirmed that MMTV was able to infect productively feline kidney cells. Since then, feline cells became increasingly used in several MMTV studies because they were known to be permissive for infection [18,19,20]. It has been also observed that a MMTV variant isolated from mice can productively replicate both in both canine and human cells by serial virus passages [21]. Recently, MMTV-like nucleotide sequences were detected in some feline and canine mammary tumours [22]. Although the aetiological role of MMTV-like virus in the induction of mammary tumours in these species has not been demonstrated yet, some studies have speculated that companion animals such as dogs and cats may represent a route of transmission of MMTV infections between those animals and human because of their intimate contacts [22,23,24,25]. Specifically, cats may transmit an adapted virus to humans. In this hypothesis, cats acquire or have acquired MMTV from mice primarily through an oral route, by feeding on mice infected with MMTV. An interesting epidemiological study from Stewart et al. [26] has reported that the incidence of human breast cancer was highest in areas where MMTV was introduced into the human population through mice. With regard to cats, Howard and Schlom [21,27] in one of their interesting experiments, have demonstrated that MMTV particles are able to infect different non-murine cell lines such as feline, canine, bat, mink, and human cells, in an easy way, and that the exogenous sequences from MMTV virus are able to acquire a broadened host range through recombination either between endogenous betaretroviruses, or different strains of endogenous or exogenous viruses; later, these findings have been confirmed by Golovkina et al. [28,29]. These studies have also demonstrated how MMTV acquires the capacity to infect and successfully establish infection in other animals and humans. Due to the confirmed role of cats in the epidemiology of a MMTV-like virus, feline mammary carcinomas were analysed to test the presence of the genetic material from the MMTV virus and to investigate the main pathological features of the positive neoplasms.

## 2. Materials and Methods

### 2.1. Animals and Tissue Samples

Twenty-four formalin fixed and paraffin embedded (FFPE) tissue samples from feline mammary tumours were retrieved from the archives of the Anatomic Pathology Service of the Department of Veterinary Medical Sciences, University of Bologna and tested for the presence of MMTV-like virus sequences and p14 protein. Seventy-eight FFPE feline mammary tumours coming from the archive of the Tumour Registry of the Department of Veterinary Science, University of Pisa, previously tested for the presence of MMTV-like virus env sequences [23], were added to the population for pathological investigations. Normal mammary gland tissues were collected from six queens died due to causes unrelated with mammary tumors. Normal tissue samples were collected during routine necropsies, after the owner’s consent. Medical history and anamnesis, included breed, age and sex, were taken for the whole population.

### 2.2. Laser Microdissection

From each paraffin block, six-micron thick sections were cut using a new microtome blade for each slide. Using a Zeiss automatic laser microdissection system (Laser Microdissection—PALM MicroBeam ZEISS Microscopy, Oberkochen, Germany), a total of 200,000–300,000 μm^2^ of epithelial cells were collected, while stromal and inflammatory cells were carefully excluded.

### 2.3. Molecular Analyses

#### 2.3.1. DNA Extraction

From each sample, four sections of 10 µm thickness were cut using microtome machine and submitted to micro dissection. Neoplastic tissue was incubated overnight in a lysis buffer containing proteinase K. DNA was extracted using Promega reagents according to the manufacturer’s protocol (Promega purification DNA FFPE). To avoid cross contamination, blank DNA samples (water) were processed in parallel with the tissue samples. The concentration of DNA was determined by dsDNA BR assay on Qubit 3 instrument (Invitrogen, Thermo Fisher Scientific, Waltham, MA, USA).

#### 2.3.2. Detection of MMTV-like Env Sequences by Nested Fluorescence-PCR

Fluorescent-nested PCR was used to detect the presence of MMTV env-like sequence using primers designed based on the sequence available in GenBank (Accession No. AF243039) and designed in such a way to generate amplicons of 201 bp or less to ensure amplification of paraffin-embedded tissues. The outer primers yield a 201-bp fragment from nucleotide positions 231 to 430 of MMTV env-like, and the inner primers yield a 191-bp fragment (nucleotide positions 240 and 431). The sequences of the outer primers were:

- Forward, 5 = -GATGGTATGAAGCAGGATGG-3 =;
- Reverse, 5 = -AAGGGTAAGTAACACAGGCA-3 =.

Inner amplification was performed with the same outer reverse primer and forward primers 5 = -AGCAGGATGGGTAGAACCTA-3 =.

Both PCR amplifications were performed in a 50-μL mixture containing 5 μL of 10X Taq buffer, 4 μL of deoxyribonucleoside triphosphates (dNTPs) mixture (2.5 mM m each dNTP), 2 μL of 10 μM each unlabelled reverse primer and 6-FAM±labeled forward primer (Applied Biosystems, Milan, Italy), and 0.25 μL Takara Ex Taq DNA polymerase (Takara Biotechnology, Dalian). Input target template was 200 ng genomic DNA in the first round PCR and 2 μL of first-round PCR product in the second round. Thermocycler conditions for PCR were initiated with denaturing at 94 °C for 10 min, followed by 45 cycles of 95 °C for 45 s, 60 °C for 45 s, and 72 °C for 1 min, and this was ended with 72 °C for 10 min. The second run of PCR using inner primers followed the same conditions as those of the first run.

To exclude PCR contamination, water controls and negative DNA samples were included every five samples in each run. As positive control DNA from MMTV-positive murine cell line Mm5Mt mammary carcinoma was used. Fluorescent amplicons were analysed by capillary electrophoresis and appeared as peaks in an electropherogram. The amplicon size was extrapolated from a molecular size ladder re-suspended in PCR buffer and run in parallel. Briefly, 2 μL of PCR products from amplification rounds were mixed with 0.5 μL of LIZ labelled size standard (GeneScan™ 600 LIZ, Applied Biosystems, Warrington, UK) and 9.5 μL of formamide (Hi-Di Formamide; Applied Biosystems, Warrington, UK).

After denaturation at 95 °C for 3 min, samples were loaded onto a 3500 Genetic Analyzer and analysed using GeneMapper software, version 3.1 (Applied Biosystems, Foster City, CA, USA).

The presence of contaminating mouse DNA was excluded by performing murine mitochondrial DNA and IAP LTRs PCR, according to Robinson et al., 2010 [30].

#### 2.3.3. DNA Sequencing

PCR amplifications of positive to MMTV-like sequences were performed again using the same mixture and thermocycler conditions but replacing fluorescent primers with non-fluorescent ones. PCR products were separated by 2% agarose gel electrophoresis, and gels were stained with Invitrogen SYBR Safe DNA Gel Stain 0.05 (Life Technologies Ι Carlsbad, CA, USA) and then visualized by a UV illuminator. The bands from sample that was positive to MMTV env-like were cut and sequenced after cleaning up with the MinEluite Gel Extraction Kit (Qiagen, Venlo, Netherlands) using Big Dye Terminator mix (Applied BioSystems, Warrington, UK). Sequencing reactions were run on a 3500 Genetic Analyzer (Applied BioSystems, Foster City, CA, USA).

#### 2.3.4. Multiple Alignment and Phylogenetic Tree

The nucleotide sequences found were aligned with the positive control for MMTV, mouse mammary tumour from HeJ mice, HMTV and previously feline sequences deposited [8]. Alignment was performed using the CLC Sequence Viewer software for multiple alignment tool ClustalW package [31]. Neighbour-joining phylogenetic analysis was performed to compare the sequences found herein with UPGMA phylogenies package for phylogenetic tree and statistical analysis. The neighbour-joining phylogenetic tree was root to MMTV sequences and inferred based on the 201-nucleotide sequence of the partial env gene of MMTV. The neighbour-joining phylogenetic tree was constructed after 1000 replicates [32].

### 2.4. Histopathologic Investigations

Tissue sections of the mammary tumours were stained with haematoxylin and eosin (H&E). Microscopic diagnosis of mammary tumours was made according to Zappulli et al. [33]. Tumours displaying different features were classified according to the most pronounced histological differentiation. Histological grading was assessed following the guidelines of Mills et al. [34]. Lympho-vascular invasion, nuclear form and mitotic count was evaluated. The absence of lympho-vascular invasion, less than or equal to 5% abnormal nuclear form and a mitotic count ≤ 62 mitosis in 10 high-power fields corresponded to Grade I (low-grade carcinoma). The presence of only one feature among lympho-vascular invasion, more than 5% abnormal nuclear form and a cumulative mitotic count > 62 corresponded to Grade II (intermediate-grade carcinoma). If any 2 of 3 of the features were present, Grade III was assigned (high-grade carcinoma).

### 2.5. Immunohistochemistry

Immunohistochemistry was performed to assess the molecular phenotype of mammary carcinomas, and the positivity for p14, protein precursor of the viral envelope. To assess molecular phenotype, seven consecutive sections (4 µm) from each tumour (n = 102) were subjected to IHC using antibodies specific for oestrogen receptor (OR), progesterone receptor (PR), receptor tyrosine-protein kinase erbB-2 (c-erbB-2), cytokeratin (CK) 5/6, CK14, CK19 and protein 63 (p63) [35].

Sections were dewaxed in xylene for 5 min, and rehydrated through graded alcohols (100, 90, and 70%) and water. A peroxidase block was performed by immersion in H_2_O_2_ 0.3% in methanol for 20 min. Antigen retrieval was achieved by placing the slides in a bath of citrate buffer (pH 6), except for those labelled with anti-CK5/6, incubated with EDTA, pH 8.0, and boiling for 16 min in an 800 W microwave oven. The slides were dried at room temperature (RT) and washed with running tap water. Non-specific bindings were prevented by incubation with 10% normal horse serum for 10 min. All antibodies were incubated with the tissue sections overnight at 4 °C. Clones, dilutions and manufacturers of primary antibodies are summarized in Table 1. Sections were then incubated with 100 μL of biotinylated horse anti-mouse/rabbit IgG antibody (H+L) R.T.U. (Vectors Laboratories, CA, USA). Antibody binding was detected using a streptavidin-biotin-peroxidase kit (Vector Laboratories, Burlingame, CA, USA). Further, 3,30-diamino-benzidine (0.05% for 10 min at RT) was used as chromogen (Vector Laboratories, CA, USA). Stained slides were subsequently counterstained in haematoxylin for 50 s followed by a wash in tap water, dehydration in graded alcohols (70, 90, and 100%), and cleared with xylene. Sections were mounted in DPX (08600E; Surgipath Europe, Peterborough, UK). As a negative control, the primary antibody was replaced with an irrelevant, isotype-matched antibody to control for non-specific binding of the secondary antibody. As positive controls, normal feline uterus (for anti-OR and -PR antibodies) and feline skin (for anti-CK5/6, -CK14, -CK19 and -p63 antibodies) were used. A human poorly differentiated invasive ductal mammary carcinoma (kindly provided by P. Viacava, Department of Oncology, University of Pisa, Italy) known to react with c-erbB-2 (HER2) antibody was used as positive control for that antibody.

Either positive or negative reactions were recorded. The positivity was evaluated accordingly to the following criteria:

- Cytoplasmic labelling of >1% of the invasive tumour cells for CK5/6 and CK14 [36];
- Nuclear labelling of >5% of tumour cells for OR and PR [37];
- Nuclear labelling of >10% of tumour cells for p63 [38].

Considering the c-erbB-2 staining, samples were scored as IHC 0, IHC 1+, IHC 2+, IHC 3+ based on the membrane staining as suggested from the latest ASCO/CAP guidelines of 2018 [39] as follows:

- IHC 0: no staining is observed or membrane staining that is incomplete and is faint/barely perceptible and in ≤10% of tumoral cells;
- IHC 1+: incomplete membrane staining that is faint/barely perceptible and in >10% of tumoral cells;
- IHC 2+: weak to moderate complete membrane staining observed in >10% of tumoral cells;
- IHC 3+: circumferential membrane staining that is complete, intense, and in >10% of tumour cells.

According to the guidelines proposed by the American Society of Clinical Oncology/College of American Pathologists (ASCO/CAP) [39], only 3+ tumors were considered positive.

### 2.6. Molecular Phenotype of Feline Mammary Carcinoma

Recording the labelling of each antibody together with the others, according to the algorithm previously modified [35,40], leads to classify feline mammary tumours into fine molecular phenotypes as follows:

- Luminal-like (luminal A or luminal B): OR+ and/or PR+, c-erbB-2 + (IHC 3+) or c-erbB-2 not positive (IHC 0, 1+, 2+), regardless of CK5/6, CK14 and p63 labelling;
- c-erbB-2 overexpressing: OR-, PR-, c-erbB-2+ (IHC 3+), regardless of CK5/6, CK14 and p63 labelling;
- Basal-like defined as OR-, PR-, c-erbB-2-, CK5/6+ and/or CK14+ and/or p63 labelling;
- Normal-like defined by no label with any marker.

### 2.7. MMTV-like p14 Immunolocalization

MMTV-like p14 protein expression was determined using IHC staining previously used for human breast cancer and feline mammary tumours [23,41] and validated by FISH analysis [42]. Four-micron thick sections from all the FMCs (n = 102) and from normal mammary tissue (n = 6) were dewaxed in xylene and rehydrated through graded alcohols to water. Antigen retrieval was performed microwaving sections for 9 min in citrate/EDTA buffer (pH 7.8). No specific peroxidase activity was blocked with 3% hydrogen peroxidase for 15 min, non-specific binding prevented by incubation with normal goat serum for 10 min. Afterwards, incubation with 1:2000-diluted rabbit polyclonal antibody anti MMTV-p14 (kindly provided by Dr J. Hochman, University of Jerusalem, Israel) was performed for 2 h at room temperature. Negative controls were performed by replacing the primary antibody with an irrelevant rabbit polyclonal antibody (anti-toxoplasma). A biotin conjugated goat derived secondary antibody was applied followed by the enzyme-labelled streptavidin and substrate chromogen (rabbit specific HRP/DAB-ABC detection IHC kit, ab 64261 Abcam). Slides were counterstained with haematoxylin. Cytoplasmic/nuclear staining for MMTV-p14 were considered positivity. To assess the specify of the reaction, an injection sarcoma of cat was also included as negative control. 

### 2.8. Statistical Analysis

The Fisher exact test analysis was used to determine if there were correlation between the identification of MMTV and data from signalment, specific histological features of tumours, and molecular phenotypes. A *p*-value of less than 0.05 was considered significant.

## 3. Results

### 3.1. Clinical Data

Feline breeds of the 102 subjects with mammary lesions included European short air (n = 60), Persian (n = 8), Siamese (n = 3), Siberian (n = 1), Maine Coon (n = 1) and Devon (n = 1) were analysed. Data on the breed of 28 subjects were not available. The population was characterized by 85 queens and 8 males, while for 9 cats, information about sex was not available. The mean age of examined cats was 11.2 ± 3.2 years old, ranging from 3 to 18 years.

### 3.2. Detection of MMTV-like Env Sequences by Nested Fluorescence-PCR

**Figure 1:**
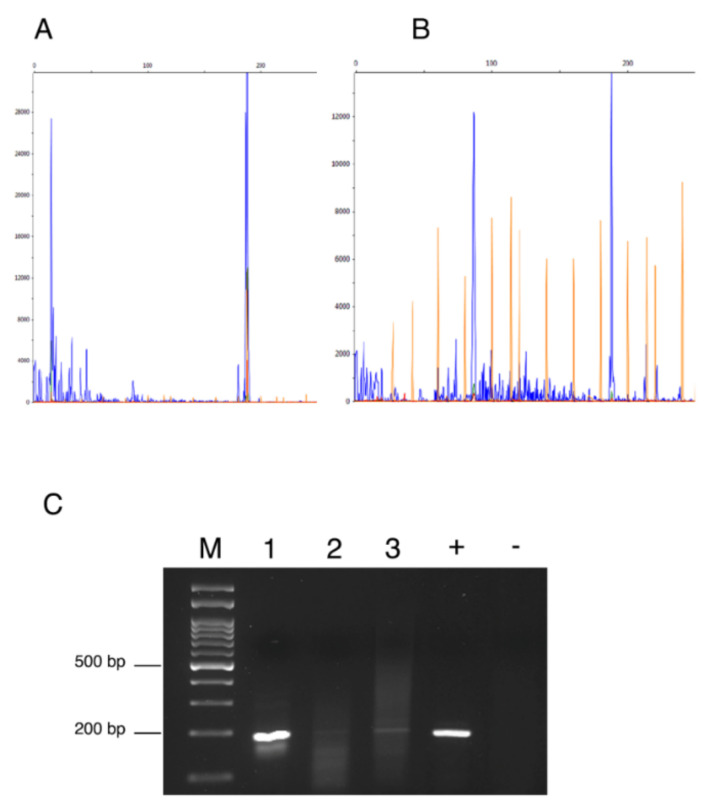
Fragment analyses showed the blue peaks at 191 bp representing the size of the amplified *env* fragment in sample ID #28983 (**A**) and sample ID #873/03 (**B**). (**C**) The 2% Agarose gel electrophoresis of PCR products with target size of 191 bp. (M): molecular weight marker; (1): ID #28983; (2): ID #259; (3): ID #873/08; (+) positive control; (−) negative control.

The extracted DNA integrity and quality were validated by the PCR amplification of specific housekeeping GAPDH gene. MMTV-like env sequences were detected in three (12.5%) out of the 24 FMCs collected in the Bologna area, analysed (Figure 1). In detail, from these three positive samples, two (ID number #28983 and #873/03) were tubulopapillary carcinomas, of which one was of Grade III and one was of Grade II, the other sample (ID number #259/00) was classified as ductal carcinoma of Grade II. None of the samples from normal feline mammary tissue were positive for MMTV-like env sequences at PCR amplification.

### 3.3. MMTV-like p14 Immunolocalization

**Figure 2:**
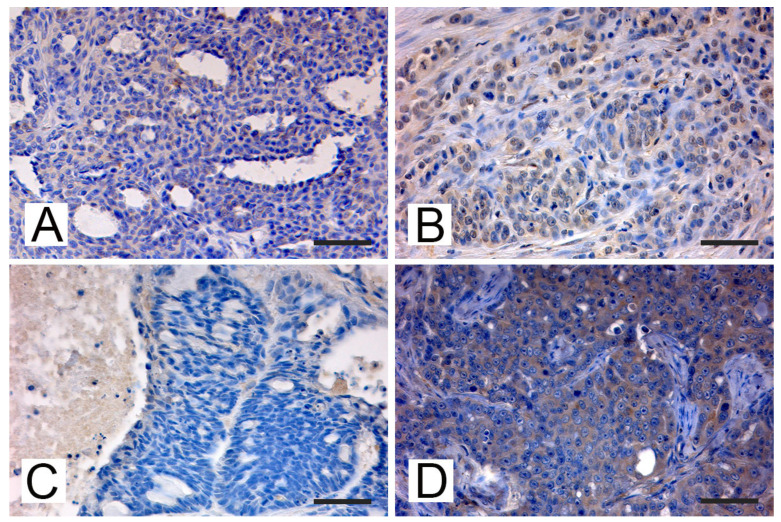
Cat. Mammary gland. Immunohistochemical expression of p14 MMTV env protein: (**A**) FMC # 84589 from Pisa, tubulopapillary carcinoma. Weak cytoplasmatic immunostaining in some neoplastic epithelial cells (bar = 50 μm); (**B**) FMC #873/08, from Bologna, tubulopapillary carcinoma. Diffuse medium intense cytoplasmic and nuclear staining was detected in some neoplastic epithelial cells that were arranged cords and tubular structures and showed moderate to severe anisocytosis and anisokaryosis, with margination of the chromatin and prominent nucleoli. Mitoses are also present (bar = 50 μm); (**C**) FMC # 259/00, from Bologna, ductal carcinoma. Scattered weak cytoplasmic staining was detected in some epithelial cells arranged in cords and tubules surrounding lumina showing anisocytosis and anisokaryosis (bar = 50 μm); (**D**) FMC #28983, from Bologna, tubulopapillary carcinoma. Diffuse intense cytoplasmic staining of neoplastic cells arranged in tubular and papillary structures showing significant anisocytosis and anisokaryosis, margination of the chromatin and large central prominent nucleoli. Several atypical mitoses are detectable (bar = 50 μm).

The expression of MMTV-p14 protein was evaluated using an anti-MMTVp14 antibody previously validated by Mazzanti et al. in order to compare the quantitative data from PCR amplification/detection methods with the expression of protein levels [42]. By IHC analysis (Figure 2) MMTV-p14 protein expression was detected exclusively in the cytoplasm and nuclei of neoplastic mammary cells of feline mammary carcinoma previously tested PCR-positive for MMTV env-like sequences, with a predominantly cytoplasmic localization. All the samples negative at the PCR amplification, the normal mammary tissues and the negative control were scored negative for p14 immunostaining. Different patterns of immunostaining were observed, ranging from scattered stained cells (Figure 2A) to staining of numerous cells (Figure 2B) and from a weak (Figure 2C) to a stronger signal (Figure 2D). Of note, the expression patterns of p14 expression were correlated with the height of the pick at the capillary electrophoresis analysis or with the thickness of the band visualized at the gel electrophoresis. In details, the sample with ID #28983 has shown the highest pick at the fragment analysis (Figure 1A) and the thicker band at the gel electrophoresis (Figure 1C, #1), while the p14 reaction showed an intense immunoreaction in numerous tumor cells with a diffuse distribution in the tumor (Figure 2D). The sample with ID #873/08 has depicted an intermediate height-pick (Figure 1B) and an intermediate thickness-band (Figure 1C, #3), with a p14 reaction showing a medium to intense cytoplasmic and nuclear staining of numerous cells (Figure 2B). While, for the sample ID #259 the lowest pick (data not shown) and a thinner band (Figure 1C, #2) was detected, a scattered weak cytoplasmic staining for p14 was observed as well (Figure 2C).

### 3.4. Multiple Alignment and Phylogenetic Analysis of MMTV-like Env Sequences

**Figure 3:**
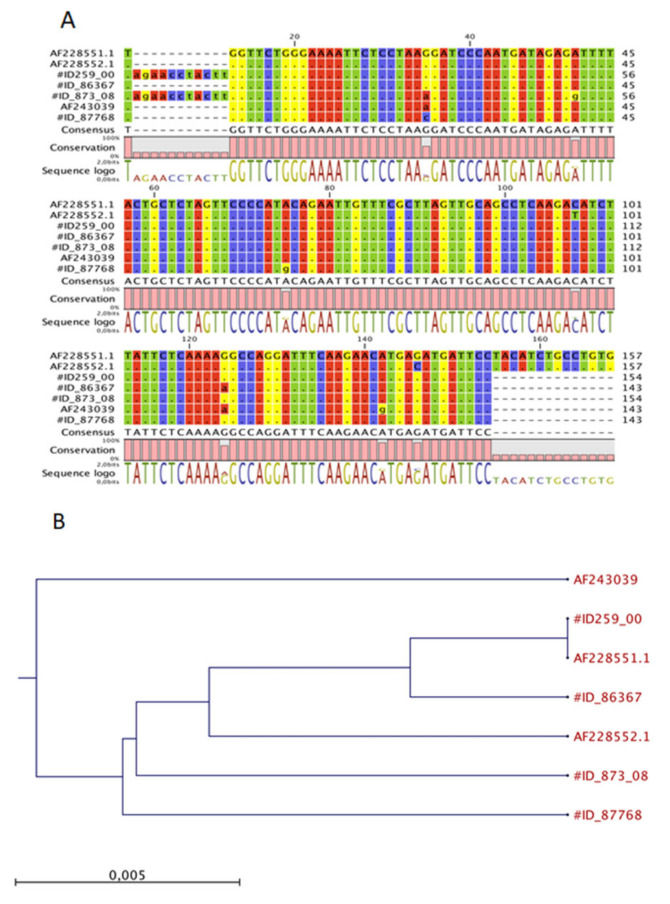
Multiple nucleotide alignment of the MMTV-like env gene sequences and phylogenetic analysis. (**A**) The env sequences from two new FMCs, #ID 28983 and #ID 873/03 respectively were aligned with NIH 3T3 (positive control for MMTV), MMTV from HeJ mice, and HMTV (Accession Numbers AF228551.1 and AF243039, respectively) and previous sequences already deposited by Civita et al. [22]; (**B**) a neighbour-joining phylogenetic tree from nucleotide sequences of env of MMTV viruses. The tree was rooted to MMTV sequences. The robustness of individual nodes of the tree was determined by using bootstrap analyses of 1.000 replicates.

The env sequences from two feline mammary tumours (indicated by their respective ID archival number) were aligned with NIH 3T3 (positive control for MMTV), mouse mammary tumour virus from HeJ mice (Accession Number AF228551.1), HMTV (Accession Number AF243039) and two feline sequences previously deposited from [8]. The results reported in Figure 3 show a sporadic nucleotide variation within the amplified env region. In particular, the new feline *env* sequences shared a of 98% and 100% to HMTV and MMTV, respectively. Moreover, no significant similarity was found when our sequences were blasted or compared to the feline and human genome sequences available in the GenBank database, thus confirming that the amplified sequences did not belong to any feline or human genomes or endogenous retroviruses.

The neighbour-joining phylogenetic analysis (Figure 3A) showed that the MMTV detected from the examined cats were classified into different clusters compared to MMTV detected in mice, humans and previously deposited feline sequences, indicating that the MMTV virus could be transmitted among these hosts (Figure 3B).

### 3.5. Histopathological Investigations

All tumour tissues collected were carcinomas, in detail—101 simple carcinomas and 1 complex carcinoma. Simple carcinomas were classified into 25 tubular carcinomas, 31 tubulopapillary carcinomas, 4 ductal carcinomas, 4 intraductal papillary carcinomas, 1 micropapillary carcinoma, 25 solid carcinomas, 9 comedocarcinomas, 1 cribriform and 1 anaplastic carcinoma. Among the simple carcinomas, 11 (11%) were of Grade I, 35 (35%) were of Grade II and 55 (54%) were of Grade III. The complex carcinoma was of Grade III. Data are summarized in Table 2.

### 3.6. Molecular Phenotype

**Figure 4:**
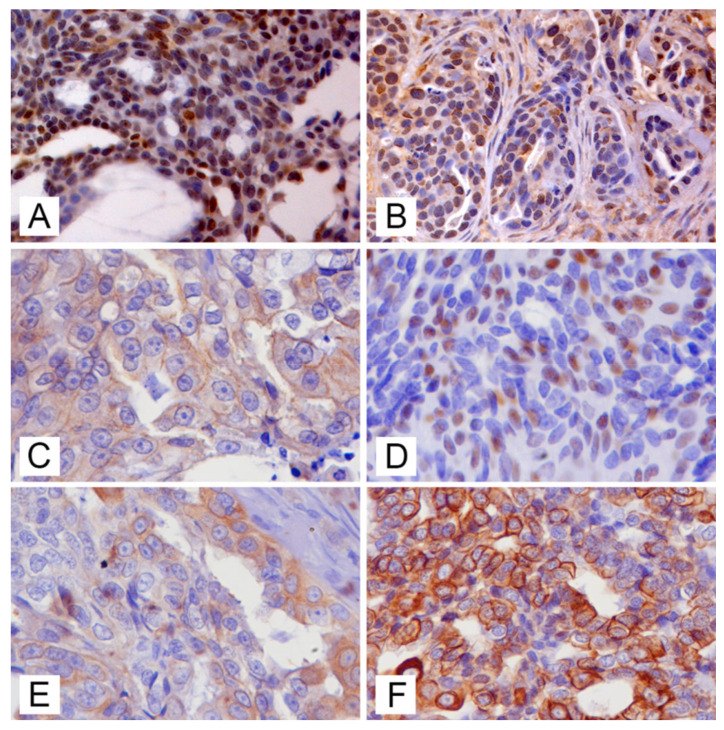
Feline mammary carcinomas (FMCs), immunohistochemistry for molecular phenotyping: (**A**) nuclear labelling for anti-oestrogen receptor (OR) antibody in a OR+ FMC (bar = 50 μm); (**B**) positive nuclear labelling for anti-progesterone receptor (PR) antibody in a PR+ FMC ((bar = 50 μm); (**C**) immunolabelling of membrane for anti-c-erbB2 antibody in a 2+ c-erbB2 FMC (bar = 50 μm); (**D**) nuclear immunolabelling for anti-p63 antibody in a p63+ FMC (bar = 50 μm); (**E**) cytoplasmic immunolabelling for anti-cytokeratin (CK) 5/6 in a CK5/6+ FMC (bar = 50 μm); (**F**) cytoplasmic immunolabelling for anti-CK14 in a CK14+ FMC (bar = 50 μm).

Molecular classification of mammary tumours was assessed in cohort of 102 feline mammary carcinomas, nine of which were positive for MMTV-like env sequences (six from Pisa, three from Bologna). Neoplastic cells staining for each antibody was evaluated and the positivity was assessed as previously described: nuclear positive staining for anti-ER (Figure 4A) and anti-PR (Figure 4B), evaluation of membrane staining for anti-c-erbB-2 (IHC 3+), nuclear positive staining for anti-p63 (Figure 4C), cytoplasmatic positive staining for anti-CK14 (Figure 4E) anti-CK5/6 (Figure 4F). Positivity or negativity for each staining were collected and recorded for each sample (data not shown). 

Following the semi-quantitative scoring system and the algorithm for molecular classification, 51 tumours (50%) were classified as luminal-like, 44 as basal-like (43%), 7 as c-erbB-2 overexpressing (7%), and no cases were null-like. Of the nine MMTV-like positive samples, six were classified as basal-like, and three were classified as luminal-like. Detailed data about MMTV-like-positive FMCs are reported in Table 3.

### 3.7. Statistical Analysis

Statistical analysis showed absence of correlation between the identification of MMTV-like sequences and p14 protein and age, sex, breed, histotype, histological grading and molecular phenotype.

## 4. Discussion

MMTV causes mammary tumours in mice and has been implicated in the aetiology of murine lymphomas [43]. Recently, the identification of MMTV-like sequences and antigens in human breast cancers and in human remains from the cooper age [15] has suggested the theory that a virus homologous to MMTV may be involved in human breast carcinomas [44]. With this hypothesis, several pieces of evidence have been accumulated through the last years. Viral particles resembling MMTV with reverse transcriptase activity were identified in human milk and neoplastic tissue of women bearing breast cancer [45,46,47,48]. Antigens related to proteins of MMTV were detected in biopsies from breast tumour and antibodies reactive with MMTV were observed in sera from patients with breast cancer [49,50,51,52,53]. MMTV sequences were identified in human breast cancer samples through hybridization analyses [54,55] and PCR amplification [12,44,56] and in other human biological materials, such as sera, saliva and milk [42,53,57,58]. Moreover, different studies suggest that the incidence of human breast cancer is geographically correlated with the natural ranges of various mice species [43] and that cats may be infected with a close homologue of MMTV [25].

In this current study, we succeeded in detecting MMTV-like sequences and MMTV p14 antigen in a different Italian feline population bearing carcinomas from and around Bologna city, in the Emilia Romagna region (Italy), confirming that these pets harbour MMTV-like sequences and p14 antigen. These data also confirmed the previous findings in a population of cats with mammary tumours from the Pisa city area, in the Tuscany region (Italy) [23]. In particular, the percentage of MMTV-like positive FMCs in the Bologna city area was higher than that detected in the previous study (12.5% vs. 7%) but lower than the ones found by Hsu et al. [22], although this study was carried out on a small cohort (12.5% vs. 22.2%). Previous studies on MMTV-induced neoplasms suggest that p14 may be involved in lymphomagenesis and mammary carcinogenesis [59]. In this study, p14 expression was confirmed in MMTV-like sequences positive samples using an IHC protocol validated by FISH analysis [42]. These findings suggest that positive cells are not only infected by the virus, but they are also capable to produce proteins useful for the virus to assembly new virions, these is an important evidence since the translational status of MMTV-like virus is required for spreading viruses via exogenous route [22]. The expression of p14 was only detectable in PCR-positive samples, while all PCR-negative samples showed no expression of the target protein. Due to this specificity, p14 expression through immunohistochemistry could be assessed as an independent method to confirm specific PCR amplifications. Other methods using to avoid false positive results were as follows: (1) the inclusion of DNA negative controls in all studies, (2) retesting of positive samples, (3) testing the amplification of murine DNA and (4) the direct sequencing of PCR fragments (or restriction fragment in case of samples with low DNA yields). Two of three samples were successfully sequenced. Sequences were highly similar to the MMTV env gene and showed in one case 98% of homology and in one case 100% homology with murine MMTV. No murine DNA was amplified from these samples. Moreover, the phylogenetic relationship was further analysed. As previous authors observed [22,23], the neighbour-joining phylogenetic tree showed that env sequences of this study were in different clusters from env sequences of humans and mice, suggesting that MMTV can transmit between these hosts. Furthermore, the new sequences were aligned with previously analysed sequences from Civita et al. [23] which had been demonstrated to not belong to FcERV and HERV-K family, so the same conclusion can be applied to them.

Considering the histopathological classification of the positive FMCs, it was not possible to directly compare morphological features of feline mammary carcinomas with the ones of human breast cancer and mouse mammary cancer. In some way, the feline tubulopapillary carcinoma, particularly the low-grade carcinoma, resembles the Dunn Type A mammary tumour. Indeed, it is a tumour of glandular epithelial origin, characterized by small cuboid epithelial cells arranged in single rows surrounding small to medium-sized cavities that are generally round but may be elongated and tubular [60]. On the other hand, feline tubulopapillary carcinoma of intermediate or high grade is probably more similar to the Dunn Type B mammary carcinoma, characterized by tumor cells of glandular epithelial origin arranged in cords, tubes, occasionally in solid sheets or nests, with acinar areas frequently found but without comprising most or all the sections such as in Type A. Moreover, the transition from predominantly acinar Dunn Type A to Type B tumour may be gradual, so distinction between Types A and B is often arbitrary [60]. Meanwhile, there was no specific correspondence between the classification of mammary tumours in different species as well as no significant correlation was found between the presence of viral sequences and the tumour histotype, the results of this study can be compared with the features of MMTV-like positive human breast cancers already described by Lawson and co-workers [61]. Indeed, considering the total population of MMTV-like positive both from Pisa and Bologna, the MMTV-like positive feline mammary carcinomas were in seven cases (78%) tubulopapillary carcinoma and only two cases carcinomas from different histotypes, although there is to take into account that tubulopapillary mammary carcinomas are very common in cats [33]. Apart from the morphological tumour type, these findings suggest that no correlation exist between positive FMCs to histological grading as observed in previous studies on cats [23] and humans [61]. As widely discussed by the scientific community, the classification of feline mammary tumour [33] is merely based on morphological rather than prognostic markers thus it is not possible to directly compare with the human molecular classification unless adding further molecular analysis [62]. With this aim, in this study we assessed the molecular phenotype considering the most common biomarkers used in human diagnostic assessment in order to make the results more comparable between different species, even with some limitation [62] and most importantly to establish the biological nature of the tumoral cells. Notably, gene amplification of c-erbB-2 or overexpression of its receptor in human medicine are linked to shorter disease-free intervals, increased risk of metastasis, and resistance to many types of therapy. Furthermore, c-erbB-2 status of breast tumors is clinically relevant due to the individualized therapeutic regimens with anticancer therapies targeting specifically tumor-associated gene products. In feline species, the percentage of FMCs overexpressing these receptors was higher than that in humans and suggests a possible important role of c-erbB-2 overexpression as a marker of malignancy. Due to these results, Millanta et al. [63] suggest that c-erbB-2 overexpressing FMCs can be considered as a natural model of c-erbB-2 overexpressing human breast carcinomas. Sorlie et al. [64] have supported the idea that tumours of similar anatomical origins could in fact represent distinct biological entities and that tumour subtypes could originate from different cell types. Particularly, they found breast cancer subtypes with gene expression pattern similar to luminal epithelial cells and others having the molecular profile of basal epithelial cells. Bearing in mind these considerations, it could be interesting to understand if MMTV shows a preferred tropism for luminal or basal-like phenotypes. The results of the molecular phenotype analysis of MMTV-like-positive FMCs cohort showed that the 67% were basal-like and the 33% were luminal-like, even though the correlation between env sequences and the molecular phenotype characterization was not significative. Similar results were found by Lawson et al. [61], in human positive breast cancer where the presence of viral sequences was investigated in combination with clinical prognostic indicators, such as ER, PR, c-erbB-2 and p53. Due to the consistency of the findings, further studies including a wide number of MMTV-like positive samples are needed to deeply investigate this aspect.

## 5. Conclusions

Our study demonstrated the presence of MMTV-like sequences and MMTV p14 antigen in another feline population in Italy, confirming the results of a previous study. The expression patter of the viral antigen seemed to correlate with the number of viral copies in samples tested, suggesting a translational status of MMTV-like virus in infected cells, but further studies are necessary to confirm these data. The immunohistochemical studies allowed to characterize the molecular phenotype of MMTV-like-positive FMCs which were mainly basal-like, while the remaining tumors were luminal-like, without a significant correlation between molecular subtype and MMTV-like positivity, as previously observed in human MMTV-like-positive breast cancer.

## Figures and Tables

**Table 1:** Primary antibodies used to assess molecular phenotype of the investigated feline mammary carcinomas.

Antibody	*Clone*	*Dilution*	*Manufacturer*
***OR***	6F11	1:40	Novocastra Laboratories Ltd., Newcastle upon Tyne, UK
***PR***	PR88	1:40	Biogenex, San Ramon, CA, USA
***c-erbB-2***	Polyclonal	1:250	Dako, Glostrup, Denmark
***CK 5/6***	D5/16B4	1:100	Zymed, San Francisco, CA, USA NeoMarkers
***CK14***	Ab-1 (LL002)	1:300	NeoMarkers, Fremont, CA, USA
***CK19***	BA17	1:50	Dako, Glostrup, Denmark
***p63***	4A4	1:50	Dako, Glostrup, Denmark

OR = oestrogen receptor; PR = progesterone receptor; *c-erbB-2 =* receptor tyrosine-protein kinase erbB-2; CK = cytokeratin; p63 = tumour protein p63.

**Table 2:** Pathologic features of the 102 feline mammary carcinomas examined.

	Histotype	Grading			Total
		I	II	III	
**Simple carcinoma**	Tubular	4	14	7	25
	Tubulopapillary	4	13	14	31
	Ductal	1	2	1	4
	Intraductal papillary	2	2	0	4
	Micropapillary	0	0	1	1
	Solid	0	2	23	25
	Comedocarcinoma	0	2	7	9
	Cribriforme	0	0	1	1
	Anaplastic	0	0	1	1
**Complex carcinoma**		0	0	1	1
**Total**		11	35	56	102

**Table 3:** Main histopathological features of the MMTV-like positive feline mammary carcinomas.

ID	Histotype	Grade	PR	ER	C-erbB-2	CK	p63	Phenotype
**80613**	TP	II	Neg	Neg	IHC 2+	Pos	Neg	Basal
**81074**	TP	II	Neg	Neg	IHC 1+	Pos	Pos	Basal
**84589**	TP	I	Neg	Neg	IHC 0	Pos	Neg	Basal
**86367**	S	III	Neg	Neg	IHC 0	Pos	Neg	Basal
**87537**	TP	II	Pos	Pos	IHC 1+	Neg	Neg	Luminal
**87768**	TP	I	Neg	Neg	IHC 0	Neg	Pos	Basal
**873/08**	TP	III	Pos	Neg	IHC 2+	Pos	Neg	Luminal
**259/00**	D	II	Pos	Neg	IHC 0	Neg	Neg	Luminal
**28983**	T	II	Neg	Neg	IHC 1+	Pos	Neg	Basal

TP: tubulopapillary, T: tubular; D: ductal; S: solid.

## Data Availability

A part of data presented in this study are openly available at doi:10.1371/journal.pone.0200839.

## References

[1] Willis A.M. (2000). Feline leukemia virus and feline immunodeficiency virus. Vet. Clin. N. Am. Small. Anim. Pract..

[2] Yoo J.H., Kim O. (2017). A simultaneous occurrence of feline mammary carcinoma and uterine cystic endometrial hyperplasia in a cat. Korean J. Vet. Res..

[3] Held W., Acha-Orbea H., MacDonald H.R., Waanders G.A. (1994). Superantigens and retroviral infection: Insights from mouse mammary tumor virus. Immunol. Today.

[4] Matsuzawa A., Nakano H., Yoshimoto T., Sayama K. (1995). Biology of mouse mammary tumor virus (MMTV). Cancer Lett..

[5] Ross S.R. (2010). Mouse mammary tumor virus molecular biology and oncogenesis. Viruses.

[6] Maeda N., Fan H., Yoshikai Y. (2008). Oncogenesis by retroviruses: Old and new paradigms. Rev. Med. Virol..

[7] Lawson J.S., Salmons B., Glenn W.K. (2018). Oncogenic Viruses and Breast Cancer: Mouse Mammary Tumor Virus (MMTV), Bovine Leukemia Virus (BLV), Human Papilloma Virus (HPV), and Epstein-Barr Virus (EBV). Front. Oncol..

[8] Callahan R., Smith G.H. (2000). MMTV-induced mammary tumorigenesis: Gene discovery, progression to malignancy and cellular pathways. Oncogene.

[9] Callahan R. (1996). MMTV-induced mutations in mouse mammary tumors: Their potential relevance to human breast cancer. Breast Cancer Res. Treat..

[10] Salmons B., Gunzburg W.H. (2013). Revisiting a role for a mammary tumor retrovirus in human breast cancer. Int. J. Cancer.

[11] Etkind P., Du J., Khan A., Pillitteri J., Wiernik P.H. (2000). Mouse mammary tumor virus-like ENV gene sequences in human breast tumors and in a lymphoma of a breast cancer patient. Clin. Cancer Res..

[12] Wang Y., Holland J.F., Bleiweiss I.J., Melana S., Liu X., Pelisson I., Cantarella A., Stellrecht K., Mani S., Pogo B.G. (1995). Detection of Mammary Tumor Virus ENV Gene-like Sequences in Human Breast Cancer. Cancer Res..

[13] Wang Y., Pelisson I., Melana S.M., Go V., Holland J.F., Pogo B.G. (2001). MMTV-like env gene sequences in human breast cancer. Arch. Virol..

[14] Wang Y., Pelisson I., Melana S.M., Holland J.F., Pogo B.G. (2001). Detection of MMTV-like LTR and LTR-env gene sequences in human breast cancer. Int. J. Oncol..

[15] Lessi F., Grandi N., Mazzanti C.M., Civita P., Scatena C., Aretini P., Bandiera P., Fornaciari A., Giuffra V., Fornaciari G. (2020). A human MMTV-like betaretrovirus linked to breast cancer has been present in humans at least since the copper age. Aging.

[16] Lasfargues E.Y., Lasfargues J.C., Dion A.S., Greene A.E., Moore D.H. (1976). Experimental infection of a cat kidney cell line with the mouse mammary tumor virus. Cancer Res..

[17] Vaidya A.B., Lasfargues E.Y., Heubel G., Lasfargues J.C., Moore D.H. (1976). Murine mammary tumor virus: Characterization of infection of nonmurine cells. J. Virol..

[18] Nusse R., Janssen H., de Vries L., Michalides R. (1980). Analysis of secondary modifications of mouse mammary tumor virus proteins by two-dimensional gel electrophoresis. J. Virol..

[19] Cato A.C., Weinmann J. (1988). Mineralocorticoid regulation of transcription of transfected mouse mammary tumor virus DNA in cultured kidney cells. J. Cell Biol..

[20] Indik S., Günzburg W.H., Salmons B., Rouault F. (2005). Mouse mammary tumor virus infects human cells. Cancer Res..

[21] Howard D.K., Schlom J. (1980). Isolation of a series of novel variants of murine mammary tumor viruses with broadened host ranges. Int. J. Cancer.

[22] Hsu W.L., Lin H.Y., Chiou S.S., Chang C.C., Wang S.P., Lin K.H., Chulakasian S., Wong M.L., Chang S.C. (2010). Mouse mammary tumor virus-like nucleotide sequences in canine and feline mammary tumors. J. Clin. Microbiol..

[23] Civita P., Menicagli M., Scopelliti C., Lessi F., Millanta F., Borsacchi S., Parisi F., Freer G., Pistello M., Mazzanti C.M. (2018). Mouse mammary tumour virus-like env nucleotide and p14 signal peptide are present in feline mammary carcinomas, but not in neoplastic or dysplastic canine mammary lesions. PLoS ONE.

[24] Laumbacher B., Fellerhoff B., Herzberger B., Wank R. (2006). Do dogs harbour risk factors for human breast cancer?. Med. Hypotheses.

[25] Szabo S., Haislip A.M., Garry R.F. (2005). Of mice, cats, and men: Is human breast cancer a zoonosis?. Microsc Res. Tech..

[26] Steward T.H., Sage R.D., Stewart A.F., Cameron D.W. (2000). Breast cancer incidence highest in the range of one species of house mouse, *Mus domesticus*. Br. J. Cancer.

[27] Howard D.K., Schlom J. (1978). Isolation of host-range variants of mouse mammary tumor viruses that efficiently infect cells in vitro. Proc. Natl. Acad. Sci. USA.

[28] Golovkina T.V., Jaffe A.B., Ross S.R. (1994). Coexpression of exogenous and endogenous mouse mammary tumor virus RNA in vivo results in viral recombination and broadens the virus host range. J. Virol..

[29] Golovkina T.V., Piazzon I., Nepomnaschy I., Buggiano V., de Olano Vela M., Ross S.R. (1997). Generation of a tumorigenic milk-borne mouse mammary tumor virus by recombination between endogenous and exogenous viruses. J. Virol..

[30] Robinson M.J., Erlwein O.W., Kaye S., Weber J., Cingoz O., Patel A., Walker M.M., Kim W.J., Uiprasertkul M., Coffin J.M. (2010). Mouse DNA contamination in human tissue tested for XMRV. Retrovirology.

[31] Thompson J.D., Higgins D.G., Gibson T.J. (1994). CLUSTAL W: Improving the sensitivity of progressive multiple sequence alignment through sequence weighting, position-specific gap penalties and weight matrix choice. Nucleic Acids Res..

[32] Saitou N., Nei M. (1987). The neighborneighbour-joining method: A new method for reconstructing phylogenetic trees. Mol. Biol. Evol..

[33] Zappulli V., Pena L., Rasotto R., Goldschmidt M.H., Gama A., Scruggs J.L., Kiupel M., Kiupel M. (2019). Mammary tumors. Surgical Pathology of Tumors of Domestic Animals.

[34] Mills S.W., Musil K.M., Davies J.L., Hendrick S., Duncan C., Jackson M.L., Kidney B., Philibert H., Wobeser B.K., Simko E. (2015). Prognostic value of histologic grading for feline mammary carcinoma: A retrospective survival analysis. Vet. Pathol..

[35] Brunetti B., Asproni P., Beha G., Muscatello L.V., Millanta F., Poli A., Benazzi C., Sarli G. (2013). Molecular phenotype in mammary tumours of queens: Correlation between primary tumour and lymph node metastasis. J. Comp. Pathol..

[36] Kim M.J., Ro J.Y., Ahn S.H., Kim H.H., Kim S.B., Gong G. (2006). Clinicopathologic significance of the basal-like subtypeof breast cancer: A comparison with hormone receptor and C-erbB-2/neu-overexpressing phenotypes. Hum. Path.

[37] Millanta F., Calandrella M., Bari G., Niccolini M., Vannozzi I., Poli A. (2005). Comparison of steroid receptor expression in normal, dysplastic, and neoplastic canine and feline mammary tissues. Res. Vet. Sci..

[38] Ramalho L.N.Z., Ribeiro-Silva A., Cassali G.D., Zucoloto S. (2006). The expression of p63 and cytokeratin 5 in mixedtumors of the canine mammary gland provides new insights into the histogenesis of these neoplasms. Vet. Path..

[39] McLemore L.E., Albarracin C.T., Gruschkus S.K., Bassett R.L., Wu Y., Dhamne S., Yim I., Lin K., Bedrosian I., Sneige N. (2021). HER2 testing in breast cancers: Comparison of assays and interpretation using ASCO/CAP 2013 and 2018 guidelines. Breast Cancer Res. Treat..

[40] Soares M., Madeira S., Correia J., Peleteiro M., Cardoso F., Ferreira F. (2016). Molecular based subtyping of feline mammary carcinomas and clinicopathological characterization. Breast.

[41] Mazzanti M.C., Al Hamad M., Fanelli G., Scatena C., Zammarchi F., Zavaglia K., Lessi F., Pistello M., Naccarato A.G., Bevilacqua G. (2011). A mouse mammary tumor virus env-like exogenous sequence is strictly related to progression of human sporadic breast carcinoma. Am. J. Pathol..

[42] Mazzanti M.C., Lessi F., Armogida I., Zavaglia K., Franceschi S., Al Hamad M., Roncella M., Ghilli M., Boldrini A., Aretini P. (2015). Human saliva as route of inter-human infection for mouse mammary tumor virus. Oncotarget.

[43] Cotterchio M., Nadalin V., Sauer M. (2002). Human breast cancer and lymphomas may share a common aetiology involving Mouse Mammary Tumour Virus (MMTV). Med. Hypotheses.

[44] Amarante M.K., de Sousa Pereira N., Vitiello G.A.F., Watanabe M.A.E. (2019). Involvement of a mouse mammary tumor virus (MMTV) homologue in human breast cancer: Evidence for, against and possible causes of controversies. Microb. Pathog..

[45] Moore D.H., Sarkar N.H., Kelly C.E., Pillsbury N., Charney J. (1969). Type B particles in human milk. Tex. Rep. Biol Med..

[46] Schlom J., Spiegelman S., Moore D. (1971). RNA-dependent DNA polymerase activity in virus-like particles isolated from human milk. Nature.

[47] Al-Sumidaie A.M., Leinster S.J., Hart C.A., Green C.D., Mc Carthy K. (1988). Particles with properties of retroviruses in monocytes from patients with breast cancer. Lancet.

[48] Melana S.M., Napomnaschy I., Sakalian M., Abbott A., Hasa J., Holland J.F., Pogo B.G. (2007). Characterization of viral particles isolated from primary cultures of human breast cancer cell. Cancer Res..

[49] Mesa-Tejada R., Keydar I., Ramanarayanan M., Ohno T., Fenoglio C., Spiegelman S. (1978). Detection in human breast carcinomas of an antigen immunologically related to a group-specific antigen of mouse mammary tumor virus. Proc. Nat. Acad. Sci. USA.

[50] Mesa-Tejada R., Oster M.W., Fenoglio C.M., Magidson J., Spiegelman S. (1982). Diagnosis of primary breast carcinoma through immunohistochemical detection of antigen related to mouse mammary tumor virus in metastatic lesions: A report of two cases. Cancer.

[51] Ohno T., Mesa-Tejada R., Keydar I., Ramanarayanan M., Bausch J., Spiegelman S. (1979). Human breast carcinoma antigen is immunologically related to the polypeptide of the group-specific glycoprotein of mouse mammary tumor virus. Proc. Natl. Acad. Sci. USA.

[52] Day N.K., Witkin S.S., Sarkar N.H., Kinne D., Jussawalla D.J., Levin A., Hsia C.C., Geller N., Good R.A. (1981). Antibodies reactive with murine mammary tumor virus in sera of patients with breast cancer: Geographic and family studies. Proc. Natl. Acad. Sci. USA.

[53] Tomana M., Kajdos A.H., Niedermeier W., Durkin W.J., Mestecky J. (1981). Antibodies to mouse mammary tumor virus-related antigen in sera of patients with breast carcinoma. Cancer.

[54] Axel R., Schlom J., Spiegelman S. (1972). Presence in human breast cancer of RNA homologous to mouse mammary tumour virus RNA. Nature.

[55] Vaidya A.B., Black M.M., Dion A.S., Moore D.H. (1974). Homology between human breast tumour RNA and mouse mammary tumour virus genome. Nature.

[56] Zammarchi F., Pistello M., Piersigilli A., Murr R., Di Cristofano C., Naccarato A.G., Bevilaqua G. (2006). MMTV-like sequences in human breast cancer a fluorescent PCR/laser microdissection approch. J. Pathol..

[57] Johal H., Ford C., Glenn W., Heads J., Lawson J., Rawlinson W. (2011). Mouse mammary tumor like virus sequences in breast milk from healthy lactating women. Breast Cancer Res. Treat..

[58] Nartey T.M., Moran H., Marin T., Arcaro K.F., Anderton D.L., Etkind P., Holland J.F., Melana S.M., Pogo B.G. (2004). Human mammary tumor virus (HMTV) sequences in human milk. Infect. Agents Cancer.

[59] Bar-Sinai A., Bassa N., Fischette M., Gottesman M.M., Love D.C., Hanover J.A., Hochman J. (2005). Mouse mammary tumor virus Env-derived peptide associates with nucleolar targets in lymphoma, mammary carcinoma, and human breast cancer. Cancer Res..

[60] Dunn T., Homburger F., Phiebig A.J. (1959). Morphology of mammary tumors in mice. Physiopathology of Cancer.

[61] Lawson J.S., Mazzanti C., Civita P., Menicagli M., Ngan C.C., Whitaker N.J., Hochman J., Braitbard O., Yosufi B., Glenn W.K. (2018). Association of mouse mammary tumor virus with human breast cancer: Histology, immunohistochemistry and polymerase chain reaction analyses. Front. Oncol..

[62] Hughes K., Dobson J.M. (2012). Prognostic histopathological and molecular markers in feline mammary neoplasia. Vet. J..

[63] Millanta F., Calandrella M., Citi S., Della Santa D., Poli A. (2005). Overexpression of HER-2 in feline invasive mammary carcinomas: An immunohistochemical survey and evaluation of its prognostic potential. Vet. Pathol..

[64] Sorlie T., Perou C.M., Tibshirani R., Aas T., Geisler S., Johnsen H., Hastie T., Eisen M.B., van de Rijn M., Jeffrey S.S. (2001). Gene expression patterns of breast carcinomas distinguish tumor subclasses with clinical implications. Proc. Natl. Acad. Sci. USA.

